# Supplementary material for: Development of cyclopeptide inhibitors of cGAS targeting protein-DNA interaction and phase separation
Source: Nat Commun. 2023 Oct 2;14:6132. doi: 10.1038/s41467-023-41892-5 (PMC10545747; doi:10.1038/s41467-023-41892-5)
Supplement: Supplementary file 1 — Supplementary Information [file 41467_2023_41892_MOESM1_ESM.pdf]

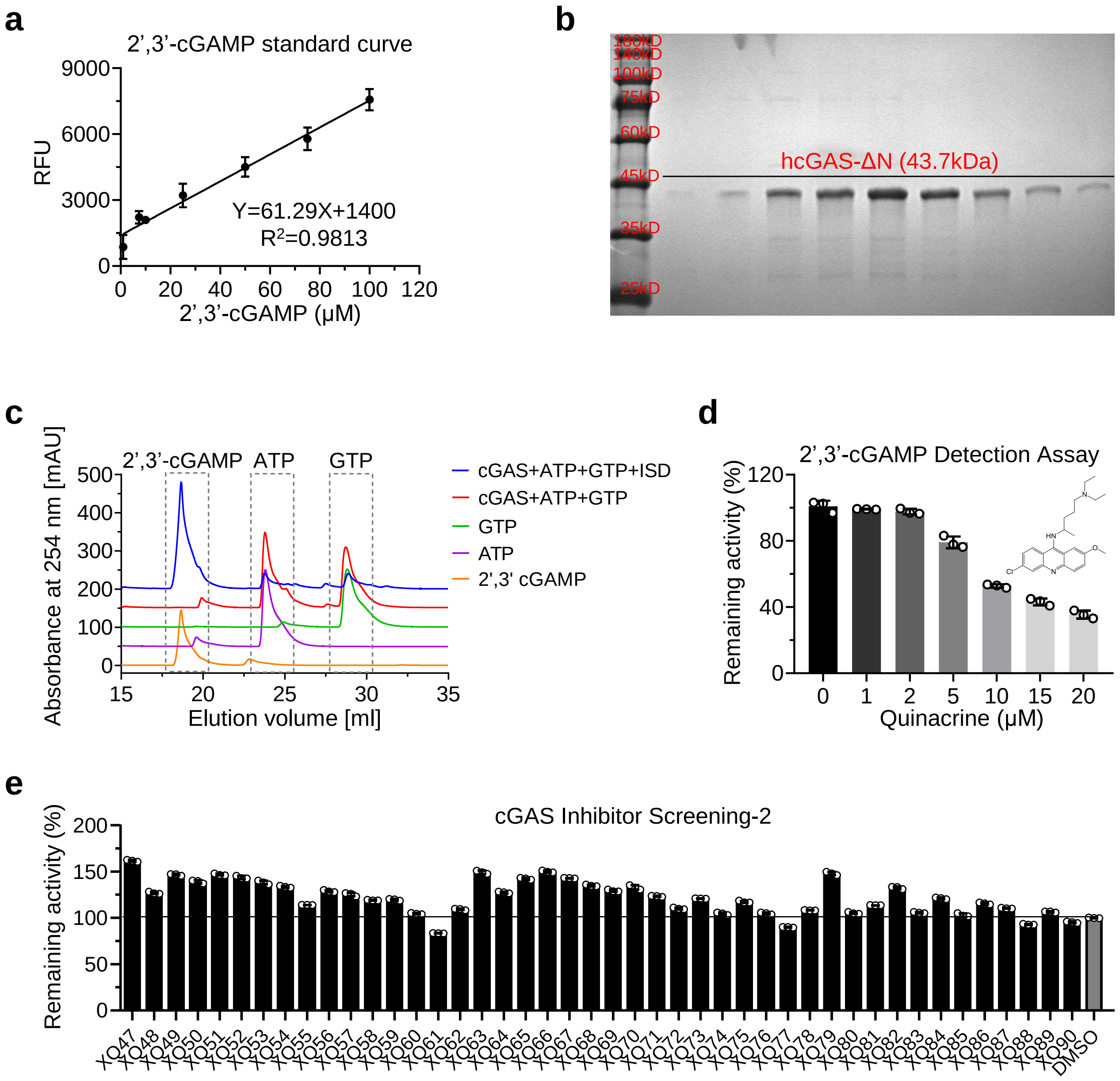

Supplementary Fig. 1. High-throughput screening of cGAS inhibitors in vitro by RNA-based fluorescent biosensor. (**a**) 2',3'-cGAMP dose curve detected by RNA-based fluorescent biosensor. (**b**) SDS–PAGE analysis of purification of N-terminally truncated human cGAS (hcGAS-ΔN, residues 147-522) in vitro. (**c**) Incubation of protein and DNA in vitro, and tested hcGAS-ΔN enzyme activity by ion exchange chromatography. (**d**) Quinacrine inhibits the synthesis of 2',3'-cGAMP in a dose-dependent manner. Remaining activity was tested by the RNA-based fluorescent biosensor for each of the indicated concentrations and normalized to no inhibitor control. (**e**) High-throughput screening for cGAS inhibitors by an RNA-based fluorescent biosensor in vitro. Screening results of XQ47-90 on the activity of purified recombinant cGAS enzyme in vitro. Data are representative of three independent experiments in (a, c, d). (Data are presented as mean ± SD, n = 3 independent samples in (a, d, e). Source data are provided as a Source Data file.

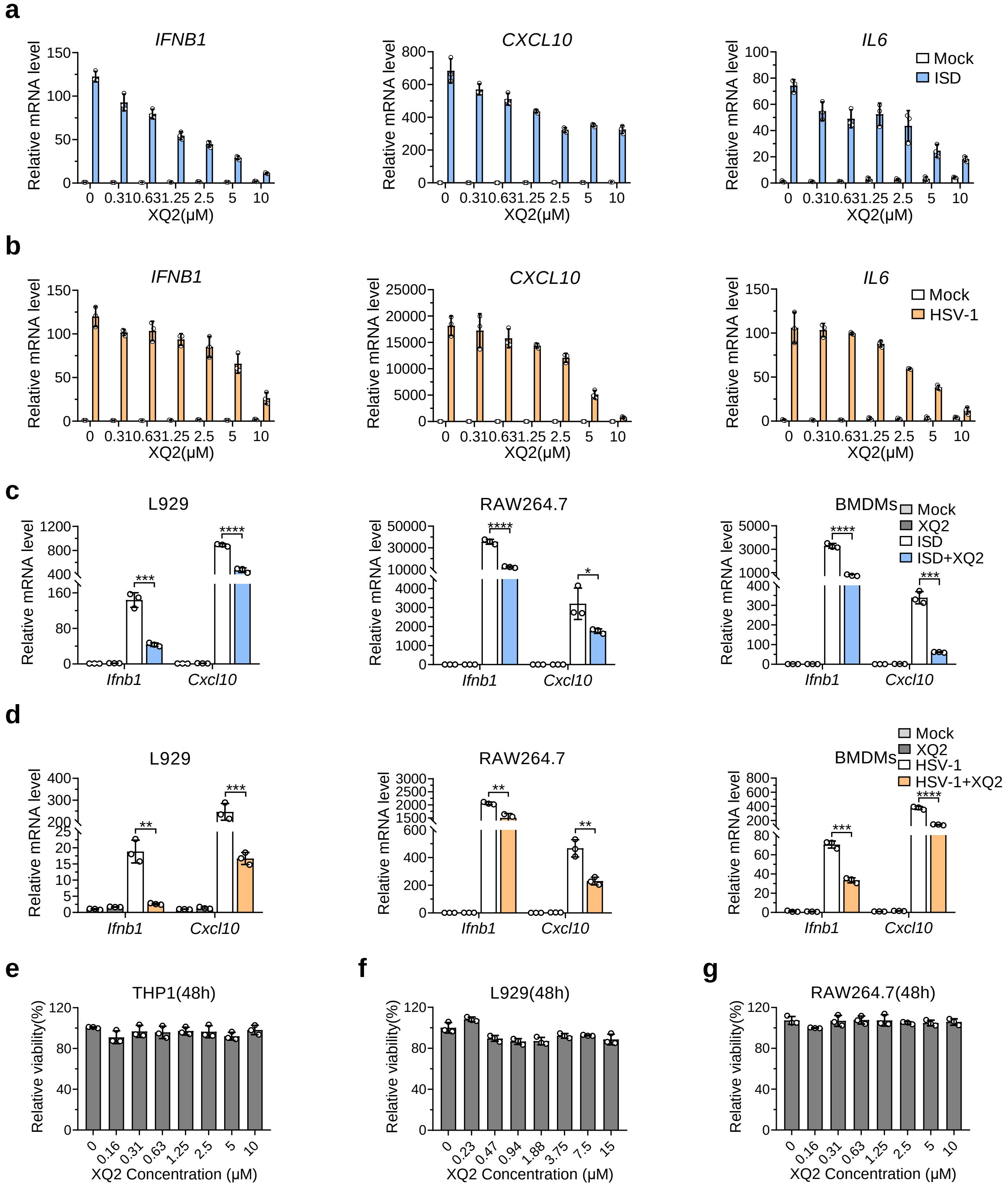
a
IFNB1
CXCL10
IL6
Mock
ISD
Relative mRNA level
XQ2(μM)
0 0.31 0.63 1.25 2.5 5 10
b
IFNB1
CXCL10
IL6
Mock
HSV-1
Relative mRNA level
XQ2(μM)
c
L929
RAW264.7
BMDMs
Mock
XQ2
ISD
ISD+XQ2
Relative mRNA level
Ifnb1
Cxcl10
d
L929
RAW264.7
BMDMs
Mock
XQ2
HSV-1
HSV-1+XQ2
Relative mRNA level
Ifnb1
Cxcl10
e
THP1(48h)
f
L929(48h)
g
RAW264.7(48h)
Relative viability(%)
XQ2 Concentration (μM)
0 0.16 0.31 0.63 1.25 2.5 5 10
0 0.23 0.47 0.94 1.88 3.75 7.5 15

Supplementary Fig. 2. XQ2 inhibits cGAS activity in human and murine derived cells. (**a**, **b**) THP1 cells were pretreated for 3 h with DMSO or the indicated doses of XQ2, and then stimulated by transfection of dsDNA for 6 h (a) or infection of HSV-1 for 8 h (b). Induction of *IFNB1*, *CXCL10* and *IL6* mRNA was measured by qPCR. (**c**, **d**) L929 cells, RAW264.7 and BMDMs were pretreated for 4 h with DMSO or XQ2 (10 μM), and then stimulated with ISD (c), or HSV-1 (d), Induction of *Ifnb1* and *Cxcl10* mRNA was measured by qPCR. (c) L929, $p = 0.0005$ (*Ifnb1*), $p < 0.0001$ (*Cxcl10*); RAW264.7, $p < 0.0001$ (*Ifnb1*), $p = 0.0406$ (*Cxcl10*); BMDMs, $p < 0.0001$ (*Ifnb1*), p = 0.0001 (*Cxcl10*); (d) L929, $p = 0.0014$ (*Ifnb1*), $p = 0.0005$ (*Cxcl10*); RAW264.7, $p = 0.0051$ (*Ifnb1*), $p = 0.0039$ (*Cxcl10*); BMDMs, $p = 0.0001$ (*Ifnb1*), p < 0.0001 (*Cxcl10*). (**e-g**) THP1 cells (e), L929 cells (f) and RAW264.7 cells (g) were exposed to the indicated doses of XQ2 for 48h, the cell viability was measured by CCK8 assay. Data are representative of three independent experiments in (a-g). (Data are presented as mean ± SD, n = 3 independent samples in (a-g), **$p < 0.01$, ***$p < 0.001$, ****$p < 0.0001$ using one-way ANOVA with Dunnett's post hoc test). Source data are provided as a Source Data file.

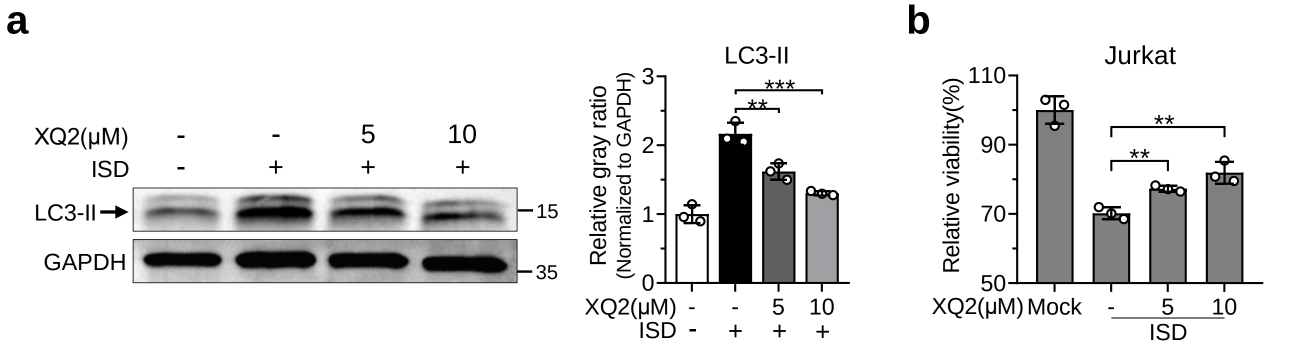

Supplementary Fig. 3. XQ2 inhibits cGAS induced autophagy and cell death. (**a**) L929 cells were pretreated for 4 h with DMSO or the indicated doses of XQ2, followed by stimulation with dsDNA for 4 h, and then cell lysates were analyzed for LC3-II and GAPDH by immunoblotting (Left); quantification of relative gray ratio (Right). p = 0.0091 (5 μM); p = 0.0008 (10 μM). (**b**) Jurkat cells were exposed to the indicated doses of XQ2, and then stimulated with ISD (4ug/ml) for 36h, the cell viability was measured by CCK8 assay. p = 0.0032 (5 μM); p = 0.0048 (10 μM). Data are representative of three independent experiments with similar results in (a), or three independent experiments in (b). Data are representative of three independent experiments in (a, b). (Data are presented as mean ± SD, n = 3 independent samples in (a, b), **$p < 0.01$, ***$p < 0.001$ using one-way ANOVA with Dunnett's post hoc test). Source data are provided as a Source Data file.

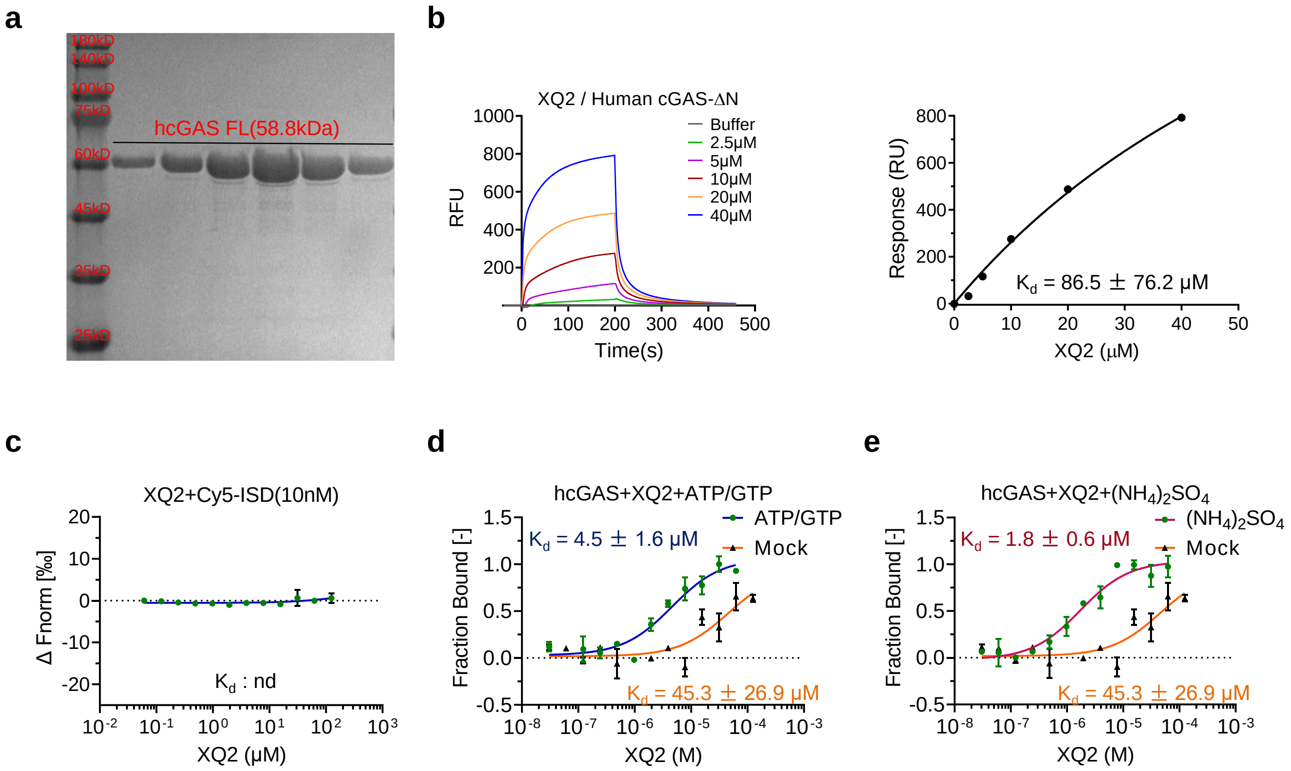

Supplementary Fig. 4. XQ2 directly binds with cGAS. (**a**) SDS–PAGE analysis of purified full-length human cGAS (hcGAS FL) in vitro. (**b**) SPR analysis of the binding of hcGAS-ΔN with XQ2. The binding affinity (K_d) was determined by fitting the binding data to a simple one-to-one binding model. (**c**) XQ2 was assessed for binding with Cy5-ISD (10nM) by microscale thermophoresis (MST). (**d**) The interaction of hcGAS with XQ2 was quantified in the presence or absence of ATP (1mM) and GTP (0.5mM) by MST. (**e**) The interaction of hcGAS with XQ2 was quantified in the presence or absence of $(NH_4)_2SO_4$ (2M) by MST. Data are representative of three independent experiments in (c-e). (Data are presented as mean ± SD, n = 3 independent samples in (c-e). Source data are provided as a Source Data file.

a

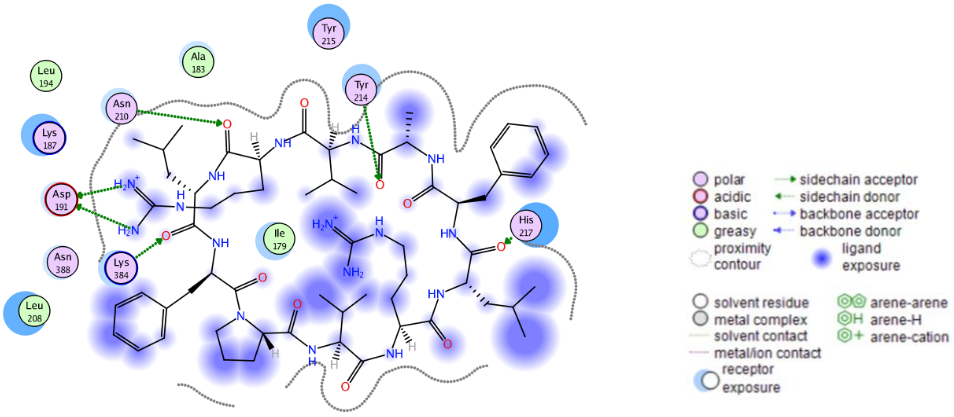

b

XQ-	Sequence	Inhibition %
46	Cyclo (AfPVLLfPVR)	56
19	Cyclo (AfPVRLfAVR)	60
8	Cyclo (LfAVRLfAVR)	52
**2**	Cyclo (LfAVRLfPVR)	40
4	Cyclo (LfPVALfPVR)	16
57	Cyclo (LfPVDAfAVR)	0
60	Cyclo (LfPVDLfPVR)	0

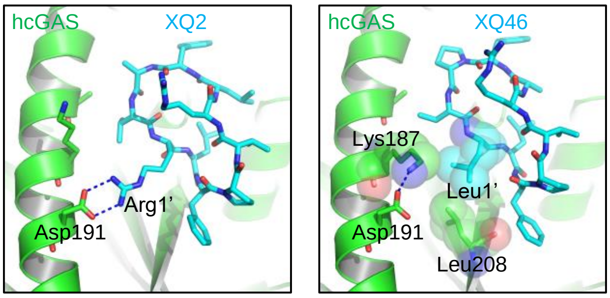

c

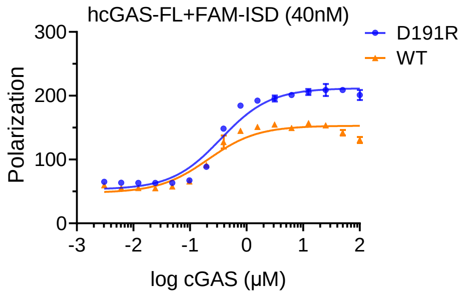

d

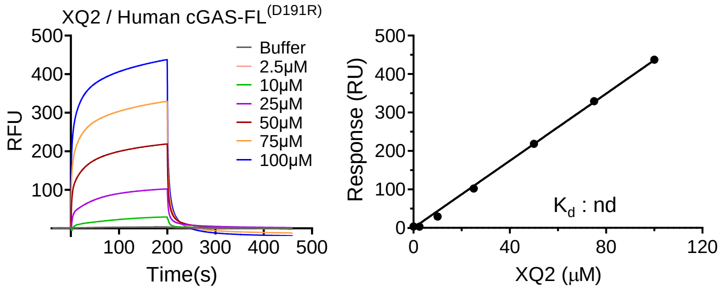

Supplementary Fig. 5. The binding site of XQ2 may locate at the cGAS-dsDNA interface. (**a**) The 2D schematic of docked structure of XQ2 with cGAS (PDB ID: 4O69). (**b**) Structure activity relationship (SAR) analysis of XQ2 a nd its analogues based on the docked structure of the peptides with cGAS. (**c**) FAM-ISD (40nM) was incubated with the indicated doses of hcGAS-FL or hcGAS-FL (D191R) mutant, and the polarization signal was detected by a microplate reader. (**d**) SPR analysis of the binding of hcGAS-FL (D191R) mutant with XQ2. The binding affinity (K_d) could not be determined by saturation binding. Data are representative of three independent experiments in (c). (Data are presented as mean ± SD, n = 3 independent samples in (c). Source data are provided as a Source Data file.

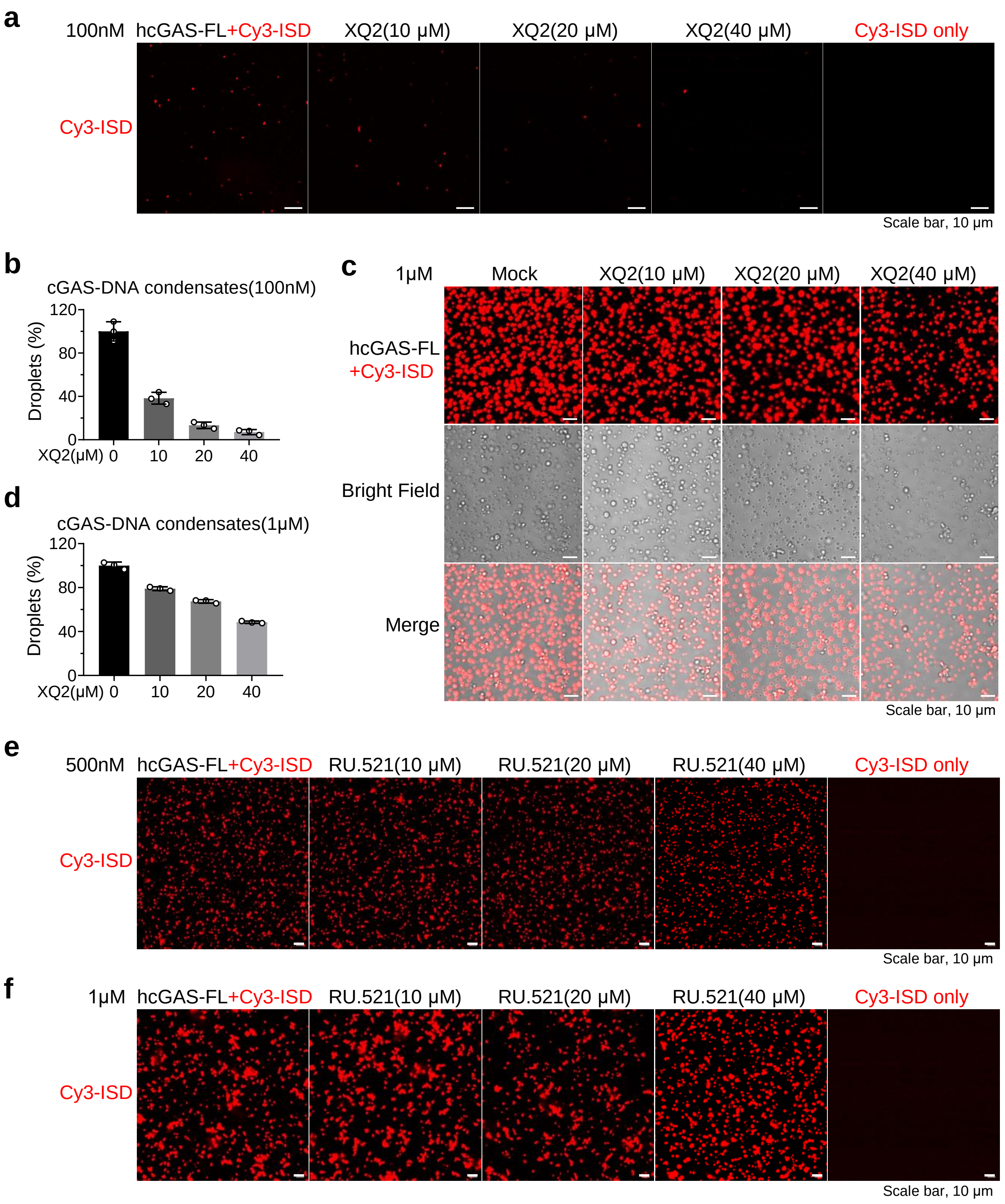
a
100nM
hcGAS-FL+Cy3-ISD
XQ2(10 μM)
XQ2(20 μM)
XQ2(40 μM)
Cy3-ISD only
Cy3-ISD
Scale bar, 10 μm
b
cGAS-DNA condensates(100nM)
Droplets (%)
120
80
40
0
XQ2(μM) 0
10
20
40
c
1μM
Mock
XQ2(10 μM)
XQ2(20 μM)
XQ2(40 μM)
hcGAS-FL
+Cy3-ISD
Bright Field
Merge
Scale bar, 10 μm
d
cGAS-DNA condensates(1μM)
Droplets (%)
120
80
40
0
XQ2(μM) 0
10
20
40
e
500nM
hcGAS-FL+Cy3-ISD
RU.521(10 μM)
RU.521(20 μM)
RU.521(40 μM)
Cy3-ISD only
Cy3-ISD
Scale bar, 10 μm
f
1μM
hcGAS-FL+Cy3-ISD
RU.521(10 μM)
RU.521(20 μM)
RU.521(40 μM)
Cy3-ISD only
Cy3-ISD
Scale bar, 10 μm

Supplementary Fig. 6. XQ2 blocks the formation of cGAS-DNA condensates in vitro. (**a**, **b**) The liquid phase condensation of hcGAS-FL (100nM) protein and Cy3-ISD (100nM) in vitro treated with DMSO or the indicated doses of XQ2 for 30 min at 37°C, and imaging analysis by confocal microscope (a). Quantification of the relative number of droplets after co-incubation (b). (**c**, **d**) The liquid phase condensation of hcGAS-FL (1 μM) protein and Cy3-ISD (1 μM) in vitro treated with DMSO or the indicated doses of XQ2 for 30 min at 37°C, and imaging analysis by confocal microscope (c). Quantification of the relative number of droplets after co-incubation (d). (**e**) The liquid phase condensation of hcGAS-FL (500 nM) protein and Cy3-ISD (500 nM) in vitro treated with DMSO or the indicated doses of RU.521 for 30 min at 37°C, and imaging analysis by confocal microscope. (**f**) The liquid phase condensation of hcGAS-FL (1 μM) protein and Cy3-ISD (1 μM) in vitro treated with DMSO or the indicated doses of RU.521 for 30 min at 37°C, and imaging analysis by confocal microscope. The images shown are representative of all fields in the well. Data are representative of three independent experiments in (a-f). (Data are presented as mean ± SD, n = 3 independent samples in (b, d). Source data are provided as a Source Data file.

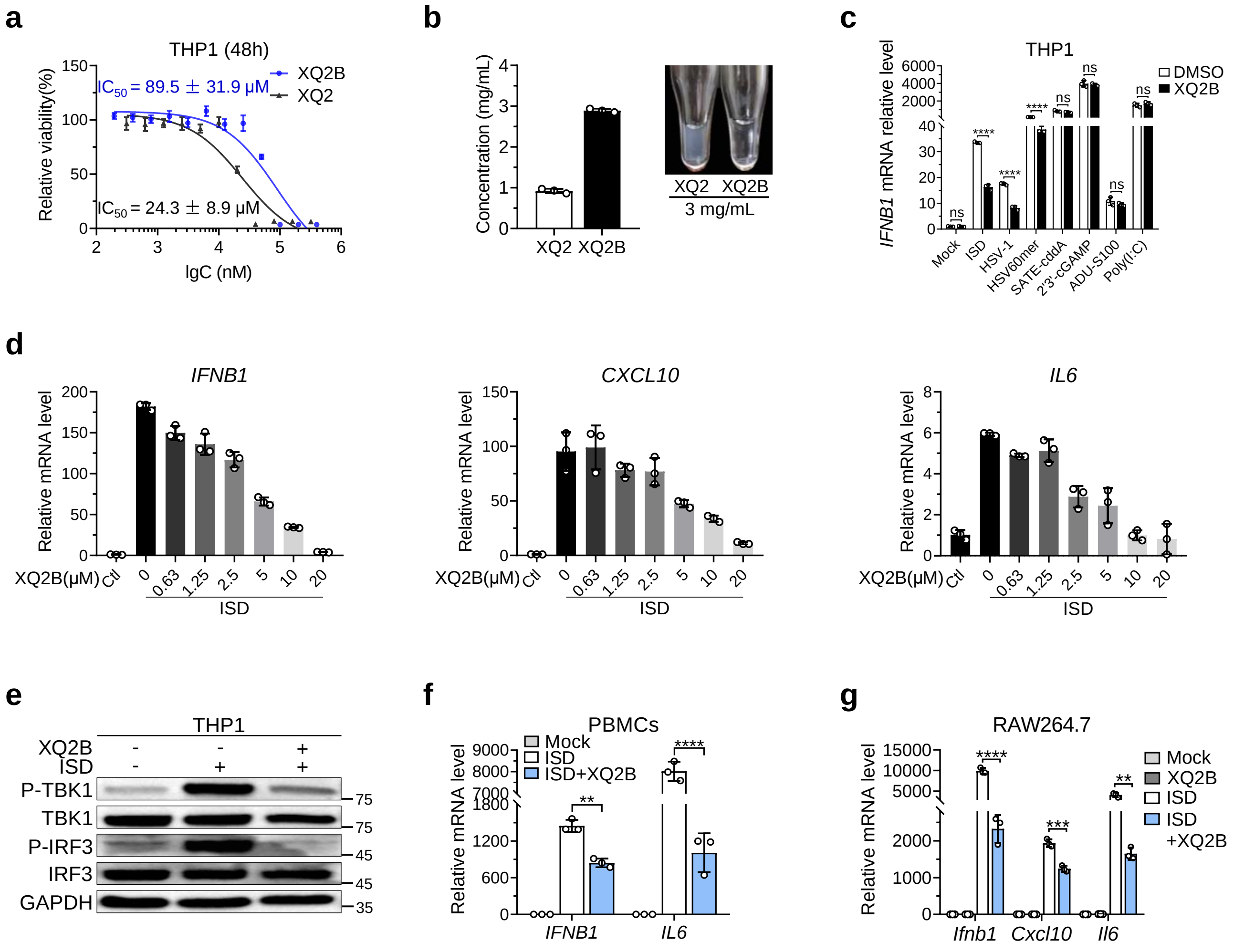

Supplementary Fig. 7. XQ2B inhibits the activity of cGAS in vitro. (**a**) THP1 cells were exposed to the indicated doses of XQ2 or XQ2B for 48h. The cell viability was measured by CCK8 assay. (**b**) The comparison of solubility of XQ2 and XQ2B. The maximum concentration of XQ2 or XQ2B dissolved in water was measured by a spectrophotometer at 280nm. The insert indicated the turbidity levels of aqueous solution of XQ2 and XQ2B in a dose of 3 mg/ml. (**c**) THP1 cells were pretreated for 4 h with DMSO or XQ2B (10 μM), and then stimulated by transfection of ISD, HSV60mer or 2'3'-cGAMP or HSV-1 infection, or addition of SATE-cddA, ADU-S100, or poly (I: C). Induction of *IFNB1* mRNA was measured by qPCR. $p < 0.0001$ (ISD); $p < 0.0001$ (HSV-1); $p < 0.0001$ (HSV60mer). (**d**) THP1 cells were pretreated for 4 h with DMSO or the indicated doses of XQ2B, and then stimulated by transfection of ISD for 6 h. Induction of *IFNB1*, *CXCL10* and *IL6* mRNA was measured by qPCR. (**e**) THP1 cells were pretreated for 4 h with DMSO or XQ2B (10 μM), followed by stimulation with ISD for 6 h, and then cell lysates were analyzed for phosphorylated IRF3 and TBK1 by immunoblotting (Left); quantification of relative gray ratio (Right). (**f**) Primary human PBMCs were pretreated for 4h with DMSO or XQ2B (10 μM), and then stimulated with ISD. Induction of *IFNB1* and *IL6* mRNA was measured by qPCR. $p = 0.001$ (*IFNB1*); $p < 0.0001$ (*IL6*). (**g**) RAW264.7 cells were pretreated for 4 h with DMSO or XQ2B (10 μM), and then stimulated with ISD. Induction of *Ifnb1*, *Cxcl10* and *Il6* mRNA was measured by qPCR. $p < 0.0001$ (*Ifnb1*); $p = 0.0008$ (*Cxcl10*); $p = 0.0017$ (*Il6*). Data are representative of three independent experiments with similar results in (e), or three independent experiments in (a-d, f, g). (Data are presented as mean ± SD, n = 3 independent samples in (a-d, f, g), ns, not significant, $^{**}p < 0.01$, $^{***}p < 0.001$, $^{****}p < 0.0001$ using one-way ANOVA with Dunnett's post hoc test). Source data are provided as a Source Data file.

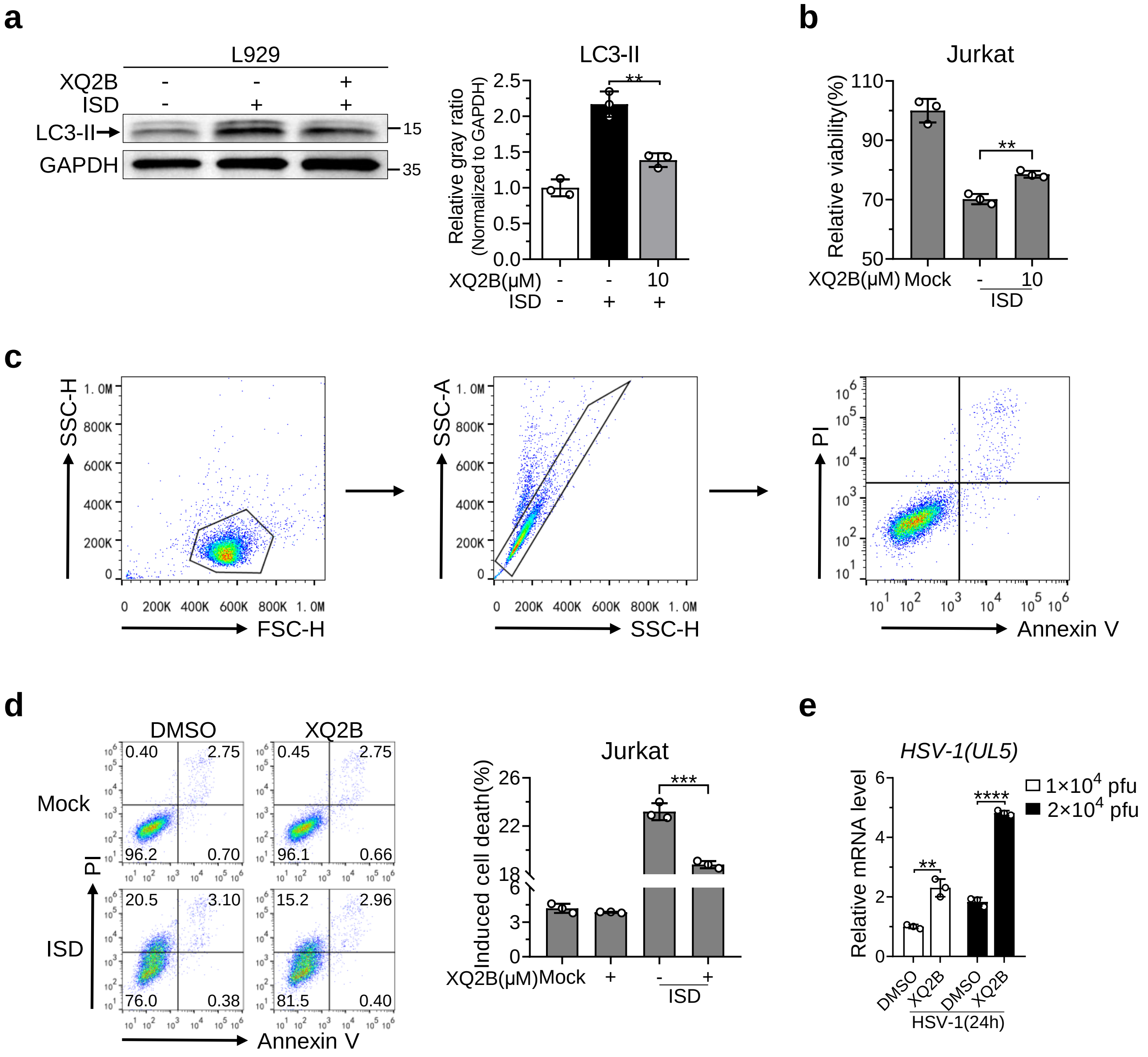

Supplementary Fig. 8. XQ2B inhibits cGAS induced autophagy and cell death. (**a**) L929 cells were pretreated for 4 h with DMSO or XQ2B (10 μM), followed by stimulation with ISD for 4 h, and then cell lysates were analyzed for LC3-II and GAPDH by immunoblotting (Left); quantification of relative gray ratio (Right). $p = 0.0026$. (**b**) Jurkat cells were pretreated with DMSO or XQ2B (10 μM), and then stimulated with ISD for 36h, the cell viability was measured by CCK8 assay. $p = 0.0022$. (**c**, **d**) Jurkat cells were treated with DMSO or XQ2B (5 μM), followed by stimulation with ISD (2ug/ml) for 4 h. Flow cytometric analysis of apoptosis assessed by Annexin V and PI staining. Analysis strategy shown was used to determine the Annexin V^+ or PI^+ population (c). Numbers in quadrants present percentages (d). $p = 0.0006$. (**e**) THP1 cells were pretreated for 4 h with DMSO or XQ2B (10 μM), and then stimulated by HSV-1 infection for indicated titer and periods. Induction of *HSV-1(UL5)* mRNA was measured by qPCR. $p = 0.0018$ (1×10^4 pfu); $p < 0.0001$ (2×10^4 pfu). Data are representative of three independent experiments with similar results in (a), or three independent experiments in (b, d, e). (Data are presented as mean ± SD, n = 3 independent samples in (a, b, d, e), $^{**}p < 0.01$, $^{****}p < 0.0001$ using one-way ANOVA with Dunnett's post hoc test). Source data are provided as a Source Data file.

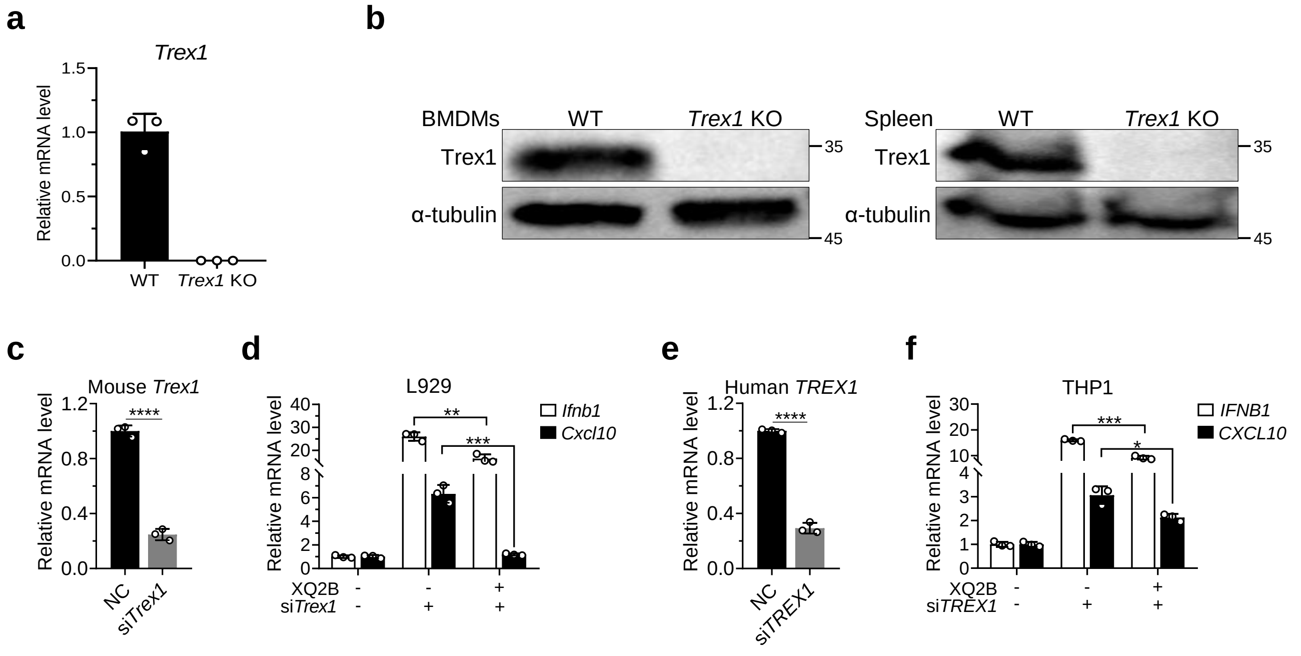

Supplementary Fig. 9. XQ2B reduces interferon expression induced by *Trex1* deficiency. (**a**) The mRNA level of *Trex1* in $Trex1^{-/-}$ BMDMs was measured by qPCR. Fold changes are relative to the WT BMDM group. (**b**) The protein level of Trex1 in BMDMs and spleen from WT mice or $Trex1^{-/-}$ mice were analyzed by immunoblotting. (**c**, **d**) *Trex1* knockdown L929 cells were treated with DMSO or XQ2B (20 μM) for 24 h, and the mRNA levels of *Trex1* (c), *Ifnb1* and *Cxcl10* (d) mRNA was measured by qPCR. (c) $p < 0.0001$, (d) $p = 0.003$ (*Ifnb1*); $p = 0.0003$ (*Cxcl10*). (**e**, **f**) *TREX1* knockdown THP1 cells were treated with DMSO or XQ2B (10 μM) for 24 h, and the mRNA levels of *TREX1* (e), *IFNB1* and *CXCL10* (f) were measured by qPCR. (e) $p < 0.0001$, (f) $p = 0.0001$ (*Ifnb1*); $p = 0.0157$ (*Cxcl10*). Data are representative of three independent experiments with similar results in (b), or three independent experiments in (a, c-f). (Data are presented as mean ± SD, n = 3 independent samples in (a, c-f), *$p < 0.05$, **$p < 0.01$, ***$p < 0.001$, ****$p < 0.0001$ using one-way ANOVA with Dunnett's post hoc test). Source data are provided as a Source Data file.

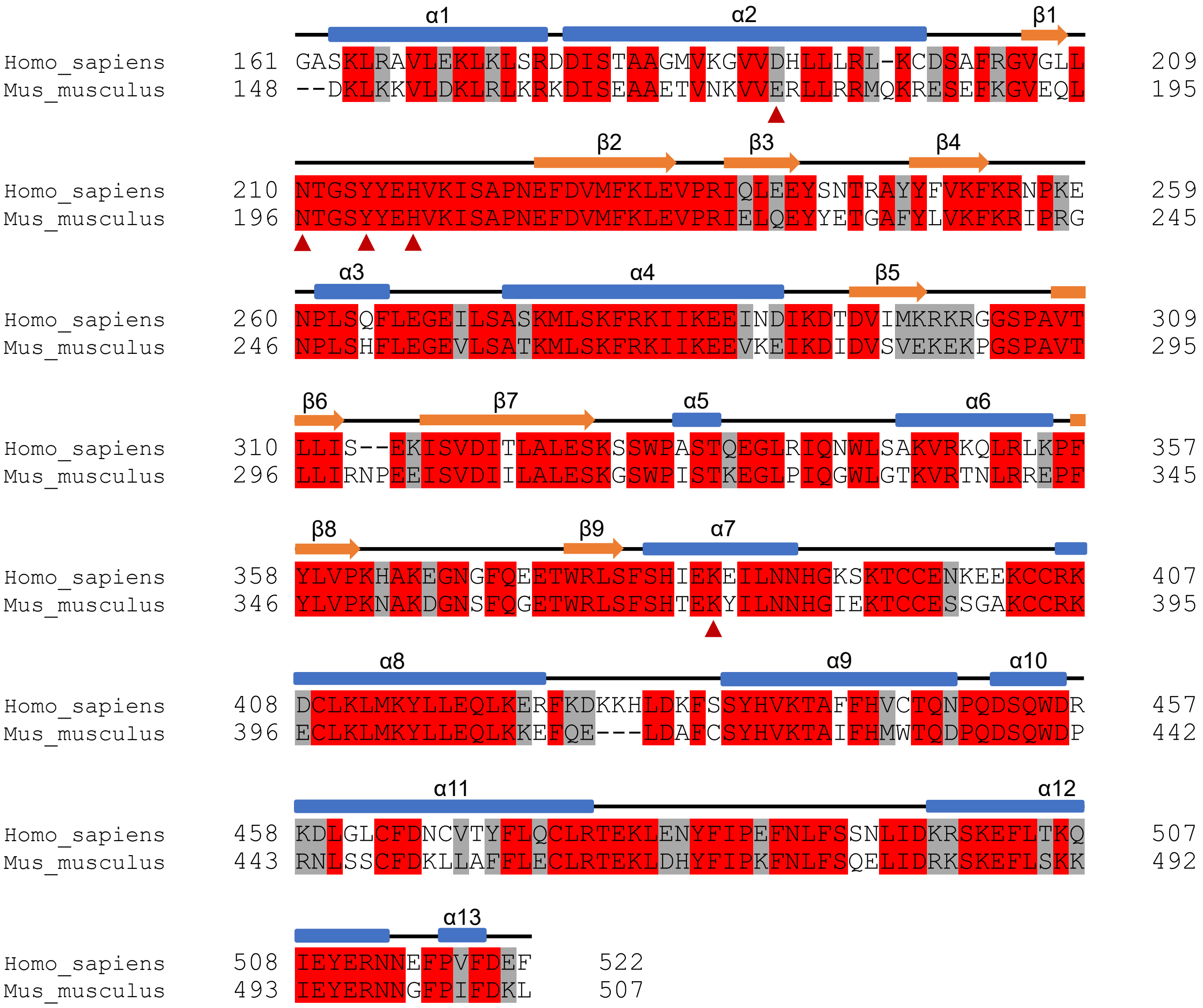

Supplementary Fig. 10 Sequence alignment of human and murine cGAS. Sequence alignment of cGAS from human and murine with highlighted conserved residues. The secondary structure from the human cGAS crystal structure is shown on top of the alignment, active site residues involved in interacting with XQ2 are indicated with red arrows.

a

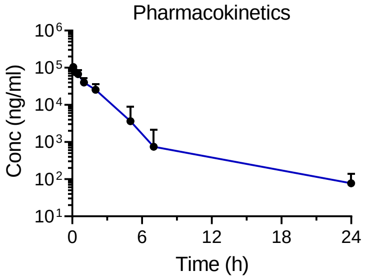

b

	T_{max}	C_{max}	AUC_{last}	AUC_{INF}	$T_{1/2}$	MRT_{last}	Cl	V_{ss}
	(h)	(ng/mL)	(h*ng/mL)	(h*ng/mL)	(h)	(h)	(mL/h/kg)	(mL/kg)
Mean	0.083	105000	135000	157000	1.1	1.2	71	95
SD	0	20300	74200	56400	0.94	0.82	27	24

Supplementary Fig. 11. Pharmacokinetics of XQ2B in C57BL/6 mice. (**a**) Pharmacokinetics in C57BL/6 mouse plasma following intravenous administration of XQ2B (n = 6, 10mg/kg/day). (**b**) Pharmacokinetics parameters of XQ2B in C57BL/6 mice (n = 6). Data are presented as mean ± SD (n = 6 mice per condition). Source data are provided as a Source Data file.

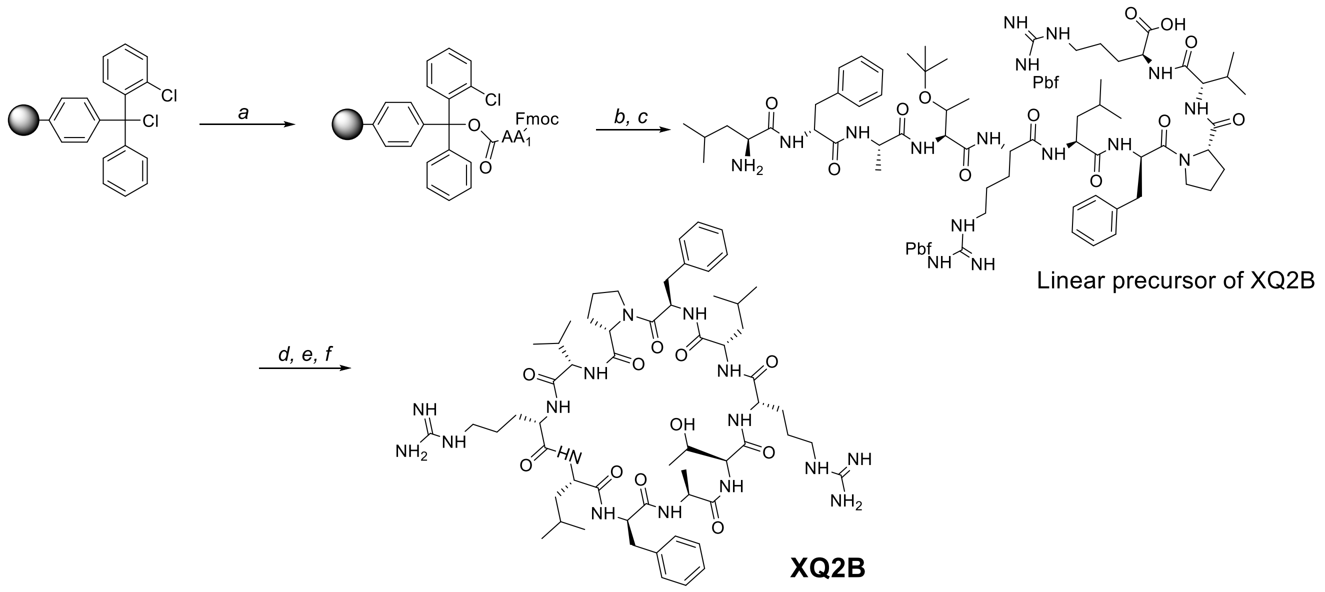

Supplementary Fig. 12. Synthesis of XQ2B. Chemical synthesis scheme depicting the synthesis of XQ2B. Reagents and conditions: a, Fmoc-AA-OH, DIEA/CH2Cl2, 3 h; b, Deprotection: 5% piperidine, 2% DBU in DMF, 15 min; c, c oupling: Fmoc-amino acid, DIC, HOBt, 3h; d, Cleavage from resin: 20% TFE in DCM; e, Cyclization: PyBOP/HOBt/DIEA (3:3:6 equiv); f, Deprotection: CF3COOH:phenol:i-Pr3SiH:H2O = 88:5:5:2, 2 h.

Supplementary Table 1. Screened library of cyclopeptides*

XQ-	Sequence	Inhibition %	XQ-	Sequence	Inhibition %
1	Cyclo (LaPVRLfPVR)	23%	46	Cyclo (AfPVLLfPVR)	56%
2	Cyclo (LfAVRLfPVR)	40%	47	Cyclo (AfPLRLfPVR)	0%
3	Cyclo (LfPARLfPVR)	33%	48	Cyclo (AfLVRLfPVR)	0%
4	Cyclo (LfPVALfPVR)	16%	49	Cyclo (AlPVRLfPVR)	0%
5	Cyclo (AfPVRLfPVR)	8%	50	Cyclo (LfPVLAfPVR)	0%
6	Cyclo (LfPVRLf(Hyp)VR)	32%	51	Cyclo (LfPLRAfPVR)	0%
7	Cyclo (LaPVRLaPVR)	0%	52	Cyclo (LfLVRAfPVR)	0%
8	Cyclo (LfAVRLfAVR)	52%	53	Cyclo (LlPVRAfPVR)	0%
9	Cyclo (LfPARLfPAR)	0%	54	Cyclo (LfPLRLfPVR)	0%
10	Cyclo (AfPVRAfPVR)	0%	55	Cyclo (LrPVALfPVR)	0%
11	Cyclo (LwPVRLwPVR)	14%	56	Cyclo (LrPVRLfPVA)	0%
12	Cyclo (Lf(Hyp)VRLf(Hyp)VR)	7%	57	Cyclo (LfPVDAfAVR)	0%
13	Cyclo (AfPARLfPVR)	7%	58	Cyclo (AfPVDLfPAR)	0%
14	Cyclo (AaPVRLfPVR)	4%	59	Cyclo (LfPTDLfATR)	0%
15	Cyclo (AfPVRLfPAR)	0%	60	Cyclo (LfPVDLfPVR)	0%
16	Cyclo (AfPARLfPAR)	1%	61	Cyclo (LwP(T(NMe))DLf(K(Dde))VR)	16%
17	Cyclo (AaPVRLaPVR)	32%	62	Cyclo (LwPTDLf(K(Dde))(V(NMe))R)	0%
18	Cyclo (AfPARAfPVR)	5%	63	Cyclo (LDfPTLfPVR)	0%
19	Cyclo (AfPVRLfAVR)	60%	64	Cyclo (DLfPTLfPVR)	0%
20	Cyclo (AfAVRAfPVR)	11%	65	Cyclo (LfPTLfPVDR)	0%
21	Cyclo (AfPVRLaPVR)	0%	66	Cyclo (LfPTLfPDVR)	0%
22	Cyclo (AaPVRAfPVR)	0%	67	Cyclo (LfPTDHfPVR)	0%
23	Cyclo (tpFlrvpFlr)	0%	68	Cyclo (LfPQDLfPVR)	0%
24	Cyclo (tpFlrtpFlr)	5%	69	Cyclo ((hex)DLfPVR(hex))	0%
25	Cyclo (fPVRfPRL)	0%	70	Cyclo (LfPTDLf(Orn(Dde))VR)	0%
26	Cyclo (PRfPRf)	0%	71	Cyclo (Lw(K(Dde))VDLf(K(Dde))VR)	0%
27	Cyclo (RfPRFP)	0%	72	Cyclo (LfPTDLf(K(Alloc))VR)	0%
28	Cyclo (RFPRFP)	0%	73	Cyclo (LwPTD(Nle)f(K(Dde))VR)	0%
29	Cyclo (FfPTRLfPTR)	7%	74	Cyclo (LwPVDLf(K(Dde))VR)	0%
30	Cyclo (VfPTRLfPTR)	7%	75	Cyclo (LwPVD(Nle)f(K(Dde))IR)	0%
31	Cyclo (LvPTRLfPTR)	30%	76	Cyclo (LfRVDLfPVR)	0%
32	Cyclo (LgPTRLfPTR)	30%	77	Cyclo (LfPVDLfRVA)	10%
33	Cyclo (Lf(Hyp)TRLfPTR)	2%	78	Cyclo (AaPVDLfPVR)	0%
34	Cyclo (LaPTRLfPTR)	0%	79	Cyclo (LfPADAfPTR)	0%
35	Cyclo (LfATRLfPTR)	0%	80	Cyclo (LfRADAfPTR)	0%

36	Cyclo (LfPARLfPTR)	11%	81	Cyclo (LfPVDAfPVR)	0%
37	Cyclo (AfPTRLfPTR)	18%	82	Cyclo (LfRVDAfPVR)	0%
38	Cyclo (LfPVRLfPVR)	0%	83	Cyclo (LfRADAfPVR)	0%
39	Cyclo (LfPV(Orn)LfPV(Orn))	3%	84	Cyclo (LfPTDAfPVR)	0%
40	Cyclo (LaPTRLaPTR)	0%	85	Cyclo (Aa(Hyp)TDLfPTR)	0%
41	Cyclo (LaP(T(Bzl))RLaPTR)	0%	86	Cyclo (Lf(Hyp)TDAfPTR)	0%
42	Cyclo (LfPTRLfPVR)	13%	87	Cyclo (AaRVDLfPVR)	0%
43	Cyclo (LfPRALfPVR)	23%	88	Cyclo (Lf(Hyp)TDAfPVR)	7%
44	Cyclo (RfPVALfPVR)	0%	89	Cyclo (AaRTDLfPVR)	0%
45	Cyclo (LfRVRLfPVA)	0%	90	Cyclo (LfPTDLfPVR)	5%

* Amino acid residues were represented by single letter code respectively unless specially mentioned, uppercase: L-amino acid residue, lowercase: D-amino acid residue. Orn: Ornithine; T(Bzl): O-benzyl-threonine; T(NMe): N-Methyl-threonine; Nle: Norleucine; Hyp: Hydroxyproline; Orn(Dde): N^{δ}-[1-(4,4-Dimethyl-2,6-dioxocyclohexylidene)ethyl]-ornithine; K(Dde): N^{ε}-[1-(4,4-dimethyl-2,6-dioxocyclohexylidene)ethyl]-lysine; K(Alloc): N^{ε}-Allyloxycarbonyl-lysine; V(NMe): N-methyl-valine; hex: 6-aminohexanoic acid.

Supplementary Table S2. List of nucleic acids used in this study

Name	Sequence (5' to 3')
**DNA oligonucleotides sequences**	
45-bp ISD	Sense: TACAGATCTACTAGTGATCTATGACTGATCTGTACATGATCTACA
	Antisense: TGTAGATCATGTACAGATCAGTCATAGATCACTAGTAGATCTGTA
45-bp FAM-ISD	Sense: FAM-TACAGATCTACTAGTGATCTATGACTGATCTGTACATGATCTACA
	Antisense: TGTAGATCATGTACAGATCAGTCATAGATCACTAGTAGATCTGTA
45-bp Cy3-ISD	Sense: Cy3-TACAGATCTACTAGTGATCTATGACTGATCTGTACATGATCTACA
	Antisense: TGTAGATCATGTACAGATCAGTCATAGATCACTAGTAGATCTGTA
HSV60mer	Sense: TAAGACACGATGCGATAAAATCTGTTTGTAAAATTTATTAAGGGTAC AAATTGCCCTAGC
	Antisense: GCTAGGGCAATTTGTACCCTTAATAAATTTTACAAACAGATTTTA TCGCATCGTGTCTTA
**RNA oligonucleotides sequences**	
Human si*TREX1*	Sense: CAAUGGUGACCGCUACGACUUTT
	Antisense: AAGUCGUAGCGGUCACCAUUGTT
Mouse si*Trex1*	Sense: GCGUCAACGCUUCGAUGACAATT
	Antisense: UUGUCAUCGAAGCGUUGACGCTT
**qPCR primer sequences for human**	
GAPDH	Sense: GGAGCGAGATCCCTCCAAAAT
	Antisense: GGCTGTTGTCATACTTCTCATGG
IFNB1	Sense: GCTTGGATTCCTACAAAGAAGCA
	Antisense: ATAGATGGTCAATGCGGCGTC
CXCL10	Sense: GTGGCATTCAAGGAGTACCTC
	Antisense: TGATGGCCTTCGATTCTGGATT
IL6	Sense: ACTCACCTCTTCAGAACGAATTG
	Antisense: CCATCTTTGGAAGGTTCAGGTTG
TREX1	Sense: CGCATGGGCGTCAATGTTTT
	Antisense: GCAGTGATGCTATCCACACAGAA
**qPCR primer sequences for mouse**	
Gapdh	Sense: TGGCCTTCCGTGTTCCTAC
	Antisense: GAGTTGCTGTTGAAGTCGCA
Ifnb1	Sense: AGCTCCAAGAAAGGACGAACA
	Antisense: GCCCTGTAGGTGAGGTTGAT
Cxcl10	Sense: CCAAGTGCTGCCGTCATTTTC
	Antisense: GGCTCGCAGGGATGATTTCAA
Il6	Sense: CTGCAAGAGACTTCCATCCAG
	Antisense: AGTGGTATAGACAGGTCTGTTGG
Trex1	Sense: CAGACCCTCATCTTCTTAGACCT
	Antisense: CAGGGCTACAGGCTTTCCC
**qPCR primer sequences for HSV-1**	
HSV-1	Sense: GGGCAGCGTTGATAGGAATTTA

	Antisense: TGTTTTCGGCGCTACCGA
HSV-1 (UL5)	Sense: ACTCGCTGATGTTGGTGTTGACGA
	Antisense: CAACGTGGCCGAGTTACTGGAAGA
**Primer sequences used for cloning of recombinant human cGAS**	
hcGAS-ΔN	Sense: CGCGGATCCCCTGTGAGCGCTCCTATC
	Antisense: CCGCTCGAGTCAAAATTCATCAAACAC
hcGAS-FL (D191R)	Sense: ATGGTTAAAGGTGTGGTTCGTCATCTGCTGCTGCGT
	Antisense: ACGAACCACACCTTTAACCATACCCGCGGCGGTAGA

Supplementary Methods

Synthesis of XQ2B

The synthesis of XQ2B is performed using a standard Fmoc/^{t}Bu solid phase procedure followed with a solution cyclization and deprotection. All amino acids used were αN-Fmoc-protected, and Fmoc-Arg(Pbf)-OH, Fmoc-Thr(^{t}Bu)-OH were used for side chain protection of Arg and Thr respectively.

Synthesis of the side chain protected linear precursor of XQ2B： 2-Chlorotrityl chloride resin (loading 0.80 mmol/g, 1.0 eq.) was swollen in CH_2Cl_2 (DCM) for 30 min. The resin was added to a round-bottle flask with a solution of Fmoc-Arg(Pbf)-OH (3.0 eq.), DIEA (10 eq.) and DCM, the mixture was drained and washed with DCM (5×5 mL) after gently agitated for 2.5 h under N_2 protection. The unreacted site on the resin was capped by a mixture of DCM /MeOH/DIEA (16:3:1, 5 mL) for 30 min. After that, Fmoc deprotection was carried out by using 5% piperidine and 2% DBU in DMF for 30 min. The resin was then washed with DMF (5×5 mL). The peptide chain elongation was performed with the mixture of Fmoc-protected amino acid (3.0 eq.), DIC (3.0 eq.) and HOBt (3.0 eq.) for 3.0 h. Kaiser test was used to check the coupling and Fmoc deprotection completeness. After the Fmoc deprotection of the last amino acid residue (Leu), The resin was washed at least 3 times with MeOH and DCM respectively. The protected linear precursor of relative cyclopeptides was cleaved with 20% TFE/DCM for 45 min. The filtrate was combined and dried in vacuo to obtain a crude product of targeted side chain protected linear precursor of XQ2B.

Synthesis of Cyclo(-Dphe-Ala-Thr-Arg-Leu-Dphe-Pro-Val-Arg-Leu-) (XQ2B): The side chain protected linear precursor of XQ2B was dissolved in cold DCM (1000 mL) to a final concentration of 0.5 mg/mL and stirred for 3 h with the presence of (PyBOP)/HOBt/DIEA (3:3:6 equiv). After solvent removal in vacuo, the cyclized peptide was treated with 5 mL of Reagent B (TFA : phenol : i-Pr_3SiH : H_2O = 88 : 5 : 5 : 2) for 2 h. The solvent was evaporated under reduced pressure, and the crude product was then precipitated by cold ether and purified by reverse-phase HPLC and lyophilized to give the product as white powders: 31.2mg, 25.0% yield; HPLC purity, 98.2%, t_R = 24.3 min. ^{1}H NMR (600 MHz, DMSO-d_6) δ 9.20 (s, 1H), 8.99 (s, 1H), 8.86 – 8.55 (m, 3H), 8.30 (d, J = 8.4 Hz, 1H), 8.01 (d, J = 8.5 Hz, 1H), 7.67 (d, J = 8.3 Hz, 2H), 7.65 – 7.62 (m, 1H), 7.46 (d, J = 33.9 Hz, 3H), 7.36 (d, J = 9.2 Hz, 2H), 7.31 – 7.22 (m, 10H), 6.97 (s, 3H), 5.00 (s, 1H), 4.89 (dd, J = 14.4, 7.1 Hz, 1H), 4.61 (dd, J = 14.2, 7.7 Hz, 1H), 4.56 (dd, J = 15.5, 7.6 Hz, 1H), 4.51 (dt, J = 14.5, 6.4 Hz, 2H), 4.37 (d, J = 6.6 Hz, 2H), 4.27 (dd,

J = 15.8, 7.6 Hz, 2H), 4.24 – 4.20 (m, 1H), 3.93 (dd, J = 11.9, 6.0 Hz, 1H), 3.18 (dt, J = 13.3, 6.2 Hz, 2H), 3.08 (dd, J = 13.8, 6.8 Hz, 2H), 2.96 (dd, J = 12.9, 5.5 Hz, 1H), 2.94 – 2.87 (m, 2H), 2.84 (dd, J = 12.8, 10.3 Hz, 1H), 2.09 (dq, J = 14.0, 6.9 Hz, 1H), 1.98 – 1.93 (m, 1H), 1.66 – 1.62 (m, 1H), 1.62 – 1.58 (m, 1H), 1.54 (dd, J = 25.8, 16.5 Hz, 4H), 1.50 – 1.41 (m, 7H), 1.38 – 1.28 (m, 4H), 1.24 (s, 1H), 1.07 (dd, J = 6.8, 4.0 Hz, 6H), 0.87 (d, J = 6.1 Hz, 3H), 0.85 (d, J = 5.9 Hz, 3H), 0.83 (d, J = 6.6 Hz, 3H), 0.79 (t, J = 5.7 Hz, 9H). ^{13}C NMR (151 MHz, DMSO-d_6) δ 172.52, 172.26, 171.84, 171.61, 171.56, 171.50, 170.81, 170.59, 170.46, 170.28, 157.12, 157.09, 137.34, 136.65, 129.76, 129.55, 128.71, 128.56, 127.35, 126.97, 68.11, 60.20, 57.80, 57.53, 56.84, 54.36, 52.28, 51.80, 50.72, 50.42, 48.38, 46.40, 41.78, 41.60, 40.91, 36.10, 31.02, 30.78, 29.98, 29.65, 25.26, 24.81, 24.69, 24.60, 23.57, 23.26, 23.14, 22.85, 19.50, 19.21, 18.87, 17.61. HRMS: calcd. for $C_{59}H_{92}N_{16}O_{11}$ $[M+2H]^{2+}$ m/z 601.3639, found, 601.3670.

The synthesis and structural characterization of XQ2 has been reported in our previous work[1].

Supplementary References

1. Chen, D. Y. et al. Dissociation of haemolytic and oligomer-preventing activities of gramicidin S derivatives targeting the amyloid-beta N-terminus. *Chem Commun* **53**, 13340-13343 (2017).
